# Fabrication of WS_2_/GaN p-n Junction by Wafer-Scale WS_2_ Thin Film Transfer

**DOI:** 10.1038/srep37833

**Published:** 2016-11-29

**Authors:** Yang Yu, Patrick W. K. Fong, Shifeng Wang, Charles Surya

**Affiliations:** 1 Department of Electronic and Information Engineering, The Hong Kong Polytechnic University, Hong Kong, China.

## Abstract

High quality wafer-scale free-standing WS_2_ grown by van der Waals rheotaxy (vdWR) using Ni as a texture promoting layer is reported. The microstructure of vdWR grown WS_2_ was significantly modified from mixture of crystallites with their c-axes both parallel to (type I) and perpendicular to (type II) the substrate to large type II crystallites. Wafer-scale transfer of vdWR grown WS_2_ onto different substrates by an etching-free technique was demonstrated for the first time that utilized the hydrophobic property of WS_2_ and hydrophilic property of sapphire. Our results show that vdWR is a reliable technique to obtain type-II textured crystallites in WS_2_, which is the key factor for the wafer-scale etching-free transfer. The transferred films were found to be free of observable wrinkles, cracks, or polymer residues. High quality p-n junctions fabricated by room-temperature transfer of the p-type WS_2_ onto an n-type GaN was demonstrated with a small leakage current density of 29.6 μA/cm^2^ at −1 V which shows superior performances compared to the directly grown WS_2_/GaN heterojunctions.

In recent years, much effort has been expended on the investigation of two-dimensional (2D) materials. In particular, graphene, with its remarkable properties such as the high conductivity and optical transmissivity, has attracted tremendous interest for potential applications in thin film and flexible electronics and optoelectronics. Unlike typical semiconductors, the most important drawback for graphene is that the material does not have a bandgap which severely limits its applications^1^,^2^,^3^. Researchers have turned to transition metal dichalcogenides (TMDCs), such as MoS_2_, WS_2_, and WSe_2_, which have drawn significant interest in recent years due to their novel layer-dependent electrical and optical properties as well as the presence of bandgaps. These materials have 2D layered crystalline structure with strong in-plane covalent bond and the out-of-plane interactions dominated by weak van der Waals (vdW) force^4^,^5^,^6^. This may potentially enable the fabrication of high quality heterojunctions which are oblivion to the lattice mismatch across the heterointerface. The wide range of 2D TMDCs with different bandgap sizes may be exploited for the development of nanoelectronic and optoelectronic devices. There have been reports on the utilization of 2D TMDC monolayers for the development of ultra-thin field-effect transistors^4^,^6^,^7^. The encouraging performances of the TMDC-based electronic and optoelectronic devices include field-effect transistors^4^,^6^,^7^, sensors^8^,^9^ and photodetectors^10^,^11^, which clearly indicate their potential applications in traditional electronic devices as well as niche applications in wearable and flexible electronics and systems^12^,^13^,^14^. Furthermore, the TMDCs have great potential as photovoltaic (PV) materials for their high absorption coefficients and proper bandgap size from 1 eV to 2.1 eV as well as the abundance and cost effectiveness of the material^5^.

Each individual layer of the TMDC material consists of a hexagonal plane of transition metal (M) atomic layer sandwiched between two chalcogen (X) atomic layers with an X-M-X structure^15^,^16^,^17^. The hexagonal structure of WS_2_ layer consists of a trigonal prism with six sulfur atoms and one tungsten atom located in the center^16^. Due to the weak interlayer forces TMDCs can be easily exfoliated by mechanical or chemical means to obtain high quality micron-sized single layer or a few layers of TMDCs. Such techniques are appropriate for fundamental investigations and the fabrication of proof-of-concept devices. However, the technique is not up scalable and hence it is not suitable for large-scale production. Chemical vapor deposition (CVD) is recognized as a large-scale chemical reaction method for large-area TMDC thin film growth for electronic and optoelectronic application^18^,^19^,^20^. van der Waals rheotaxy (vdWR) grown WS_2_ thin films were synthesized using S and WO_3_ precursors at a high temperature of 1000 °C by CVD technique on Ni-coated (5 nm) substrate. TMDCs suffer from a mixture of type I and type II crystallites, with their *c* axes parallel to and perpendicular to the substrate surface respectively as shown in Fig. 1. It is well-known that highly type II textured thin films can be grown on molten Ni promoter on sapphire substrates at high temperatures by rheotaxy which is attributed to the minimization of the film-substrate interface energy by forming molten NiS_*x*_ droplets^21^,^22^. In our experiments, the application of an ultra-thin Ni as a texture promoter is found to dramatically improve the crystal quality and carrier mobility of the WS_2_ thin film.

Most of the conductors and semiconductors become unstable at the growth temperature of WS_2_ which is as high as 1000 °C. This presents substantial barrier for the fabrication of WS_2_-based heterojunctions and thereby greatly limiting the application of the material. It has been noted that transferring the as-grown WS_2_ thin films to a suitable substrate is an effective way to circumvent this limitation. The etching-free wafer-scale transfer method reported in this paper is vital for electronics and optoelectronics applications due to its convenience for fabricating wide range of heterojunctions without concern for lattice mismatch, differences in the thermal expansion coefficient and optimization of the growth parameters on the target substrates. The transfer of graphene is one of the most widely studied topics, however the process may not be easily adopted for the transfer of WS_2_ as the technique involves the use substrate etchants, such as HF and KOH which generally induce contamination or damages on the film surface and may cause significant degradations in the device characteristics^23^,^24^. Thus, we adopted an etching-free transfer approach for the exfoliation of wafer-scale WS_2_ thin films followed by transfer to the target substrates without observable wrinkles, cracks and polymer residues. This approach greatly enhances the potential for applications of WS_2_ thin films in electronic and optoelectronic devices and systems.

In the following sections we will report a systematic investigation of the growth of high quality WS_2_ films by CVD both on sapphire and directly on an n-type GaN layer. Detailed studies on wafer-scale exfoliation and etching-free transfer of WS_2_ were also conducted. The results demonstrate that through careful optimization of the growth and etching-free transfer process, wafer-scale transfer of WS_2_ layers, with no observable cracks or wrinkles, can be achieved. Furthermore, we demonstrate the fabrication of high quality WS_2_-based p-n junctions with substantially reduced leakage current density compared to the devices by direct growth of WS_2_ thin films on an n-type semiconductor substrate.

## Results and Discussion

### CVD Growth of WS_2_ Thin Film

Sulfurization of thin WO_3_ layer requires high energy to replace the O atom by S atom and to change the crystal structural from the WO_3_ to WS_2_, so high sulfurization temperature (800 °C–1000 °C) is needed for our CVD growth of WS_2_. Generally, low-temperature grown WS_2_ exhibits small random crystallites with their c-axis parallel or perpendicular to the substrate surface. On the other hand, high sulfurization temperature favors the larger crystal layers with their c-axis perpendicular to the substrate surface. Sapphire is a substrate of choice for the growth of WS_2_ due to its chemical inertness and temperature stability. Also, sapphire has similar thermal expansion coefficients with WS_2_ and thus avoids developing large strains at the interface during material growth at a high temperature of 1000 °C^25^. We performed detailed investigations on three types of WS_2_ films: i.) WS_2_ films grown on sapphire substrate without using the Ni texture promoter (type A); ii.) WS_2_ films grown on sapphire with the use of a 5 nm thick Ni texture promoter (type B); and iii.) WS_2_ film grown on n-type GaN thin films with the use of a 5 nm thick Ni texture promoter (type C). The surface morphologies of the different types of WS_2_ thin films were analyzed by SEM technique. Figure 2 shows the SEM images for the three types of WS_2_ films. Type A film exhibited high concentration of micro cracks on the film surface which is attributed to the weak interaction between the different crystal planes. In Fig. 2a, large number of randomly oriented type-I textured crystallites were observed on the surface of the film. However, type B vdWR grown WS_2_ thin films using Ni as the texture promoter, on the other hand, were much more uniform with large layered type-II crystallites and had few crystallites scattered on the surface which can be observed from the cross-sectional SEM image as shown in Fig. 2b. In a thermally activated process, the surface diffusion of the adatoms is suppressed at low growth temperatures. This results in many nanocrystallites and the dangling bonds on the prismatic face of these nanocrystallites are highly reactive. As a consequence, these dangling bonds are bonded to the substrate to form type-I textured WS_2_. Compared to type A film, carrier mobility of type B film was remarkably increased from 3.25 cm^2^/Vs to 63.3 cm^2^/Vs with the bulk carrier (hole) concentration of 7.12 × 10^16^ cm^−3^. The improvement in the crystallinity of the type B film is attributed to the use of Ni texture promoter which facilitates the formation of liquid NiS_x_ phase, with a melting point of 637 °C^21^,^22^, that exists at the grain boundaries during the sulfurization process. It is well known that rheotaxy is a growth technique based on a molten substrate. The liquid NiS_x_ droplets may serve as nucleation sites for liquid epitaxy resulting in the distinct horizontal growth of WS_2_ crystallites with a substantially decreased level of micro-strain and defects. Type C film consists of compact small crystallites. Although both the GaN and sapphire substrates have similar lattice constant and thermal expansion coefficients compared to the WS_2_ film, the thermal stability of sapphire at a high temperature of 1000 °C is significantly better than GaN. The sapphire substrate is chemically inert and has good thermal stability at high temperature above 1000 °C^26^. However, degradation of the crystalline quality and surface morphology of the GaN layer when annealed at 1000 °C had been revealed by Raman and photoluminescence (PL) spectrum^27^, which is believed to be the key underlying factor for the poor crystalline quality for WS_2_ thin film deposited on GaN. Thus, the transfer of p-type WS_2_ onto an n-type substrate will be the preferred technique for the fabrication of p-n junction devices because this provides a wider selection of n-type materials including those that may not sustain the high sulfurization temperature.

Figure 3a and b show the high resolution X-ray diffraction (HXRD) patterns for type A and type B films, respectively. Four strong diffraction peaks located at 14.4°, 28.9°, 43.9° and 59.8° corresponding to the (002), (004), (006) and (008) crystal planes of WS_2_ respectively are observed for the type B film. However, there are only two main peaks for the type A film with much lower intensity. It shows that only the (002) family diffraction peaks was detected in the HXRD for type B film, revealing strong preferential growth along the [001] direction. The full width at half maximum (FWHM) of (002) peak for the type A and type B films were 0.565° and 0.188°, respectively. This indicates substantial enhancement in the film quality for type B film. It is confirmed, through HXRD measurement, that no NiS_x_ phase-related diffraction peak was found in the film, indicating that the volume of the NiS_x_ phase in the WS_2_ film is very low. Minor residual NiS_x_ phase can only be detected by EDX and the EDX results can be found in the Supplementary Fig. S6 and Table S1. It was reported that the residual NiS_x_ phases distributed at the grain boundaries do not strongly impact on the electrical properties of the WS_2_ film^21^. Figure 3c shows the intensity of the HXRD pattern for type C film which is much lower than either type A or type B films. The FWHM of type C (002) WS_2_ peak is 0.254° which is 35% wider than that of type B film, indicating lower crystallinity for type C film.

### Wafer-Scale Transfer of WS_2_ Thin Film

Metal oxides such as Al_2_O_3_ are typically hydrophilic. It is because the Al atoms at the surface of Al_2_O_3_ are electron deficient and form hydrogen bonds with interfacial water molecules^28^. However, WS_2_ or MoS_2_ crystal planes are chemically inert with low surface energy and exhibit hydrophobic nature^29^. Exploiting the different surface properties, water molecules can be induced to penetrate into the interface between polymer coated WS_2_ thin film and sapphire substrates leading to a driving force to separate the WS_2_ thin film from the sapphire. Using this technique, the WS_2_ thin film can be exfoliated from the sapphire substrate and transferred to any other flat substrate without any degradation in the film quality. It is noted that type A film cannot be exfoliated in wafer-scale form due to the coexistence of type I and type II crystallites, which will be discussed in details later in this section. The etching-free transfer method has the advantage of maintaining good integrity of the film without inducing any cracks or wrinkles in contrast to the conventional wet chemical etching transfer method adopted in the transfer of graphene^30^,^31^. Figure 4 illustrates the following: (a) an as-grown type B WS_2_ thin film deposited on a two-inch sapphire wafer; (b) a separated polystyrene polymer-coated WS_2_ thin film floated on water surface; (c) WS_2_ thin film transferred to a four inch SiO_2_/Si wafer; and (d) optical image of WS_2_ film after being transferred to the SiO_2_ and exhibited high integrity, uniformity and no observable wrinkles or cracks. Complete transfer of the entire WS_2_ film from the sapphire substrate with high integrity can be achieved using this technique. No residue WS_2_ material was found on the substrate as confirmed by the optical microscope and Raman spectrum which is a highly sensitive technique capable of detecting even a monolayer of residue material. This enables the reuse of the substrates for the WS_2_ thin film growth.

Both the XRD peak position and intensity for WS_2_ were nearly the same before and after layer transfer to a target substrate. In addition, the FWHM of (002) WS_2_ before and after layer transfer were 0.190° and 0.188° respectively. Similar Hall mobility was maintained for type B film after transferring to an arbitrary substrate. This is attributed to the fact that our process did not involve any corrosive chemical etchants which enables the reuse of the substrates for the WS_2_ thin film growth. The XRD pattern for a typical WS_2_ thin film grown on the reused sapphire substrate had nearly the same intensity and FWHM of 0.189°. The Hall mobility was found to be as high as 62.1 cm^2^/Vs which is highly comparable to the WS_2_ thin films grown on fresh sapphire substrates of mobility around 60 cm^2^/Vs.

The speed of the exfoliation process can be well controlled by adjusting the thickness and hardness of the polystyrene polymer. This was done by controlling the concentration of the polymer, the speed of the spin coating process and the pre-baking time. Using the optimized thickness and hardness of the polystyrene polymer, the time required for a complete exfoliation of a two-inch wafer was reduced to a few minutes. Figure S5 in Supplementary Information illustrates the wafer-scale WS_2_ layer transfer process using polystyrene by an etching free method. It is stressed that only type B WS_2_ can be exfoliated in wafer-scale. The existence of type I crystallites in type A WS_2_ hinders the penetration of water molecules at the film-substrate interface. For the conventional wet chemical etching transfer method, the chemical etchants, usually HF or KOH, will corrode or contaminate the film surface with serious implications in the electrical and optoelectronic properties of the device. However, in our etching-free transfer process, the WS_2_ thin film was stable in water which enables a less intrusive exfoliation of the WS_2_ thin films from the sapphire substrates.

Another key point for the exfoliation process is that the adhesive force of the polystyrene polymer on the WS_2_ thin film should be stronger than the interaction between the WS_2_ thin film and the sapphire substrate. If the adhesive force is weak, the WS_2_ thin film will not be completely separated by the water penetrated between the WS_2_ thin film and the substrate inducing significant cracks in the film during the exfoliations. By careful optimization of the pre-baking time of the polystyrene polymer, an optimal adhesive force for integrated exfoliation can be achieved.

The hydrophobic property of the carrier polymer is an important factor for exfoliation without cracks and wrinkles. The polymer-coated WS_2_ thin film after separation from the substrate was quite soft and can be easily broken or folded together. Each fold will leave a wrinkle after the transfer. The high hydrophobicity of the polymer excludes any water droplet on the top surface of the polymer coated WS_2_ thin film when the film floats on the water surface after separation from the substrate. This allows the film to stretch out automatically on the water surface as shown in Fig. 4b, which effectively eliminate the wrinkles during the transfer process. The polystyrene polymer has a better hydrophobic property than the PMMA polymer^32^ and it is more effective in stretching out the WS_2_ film on the water surface. It is noted that using PMMA polymer as the carrier polymer will lead to the presence of wrinkles on the WS_2_ surface after being transferred to the target substrate whereas using polystyrene polymer as the transfer agent will effectively eliminate all wrinkles in our experiments.

**Figure 1:**
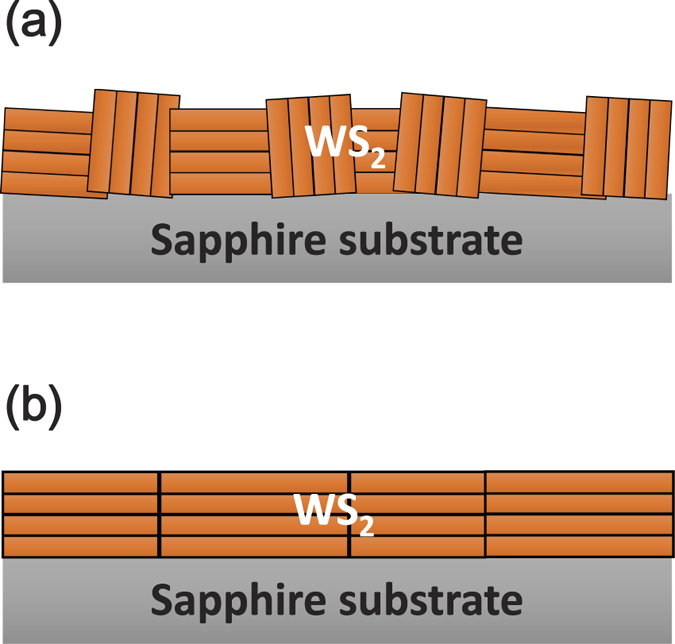
Schematic diagram showing (**a**) mixture of type I and type II and (**b**) pure type II textured WS_2_ crystallites.

**Figure 2:**
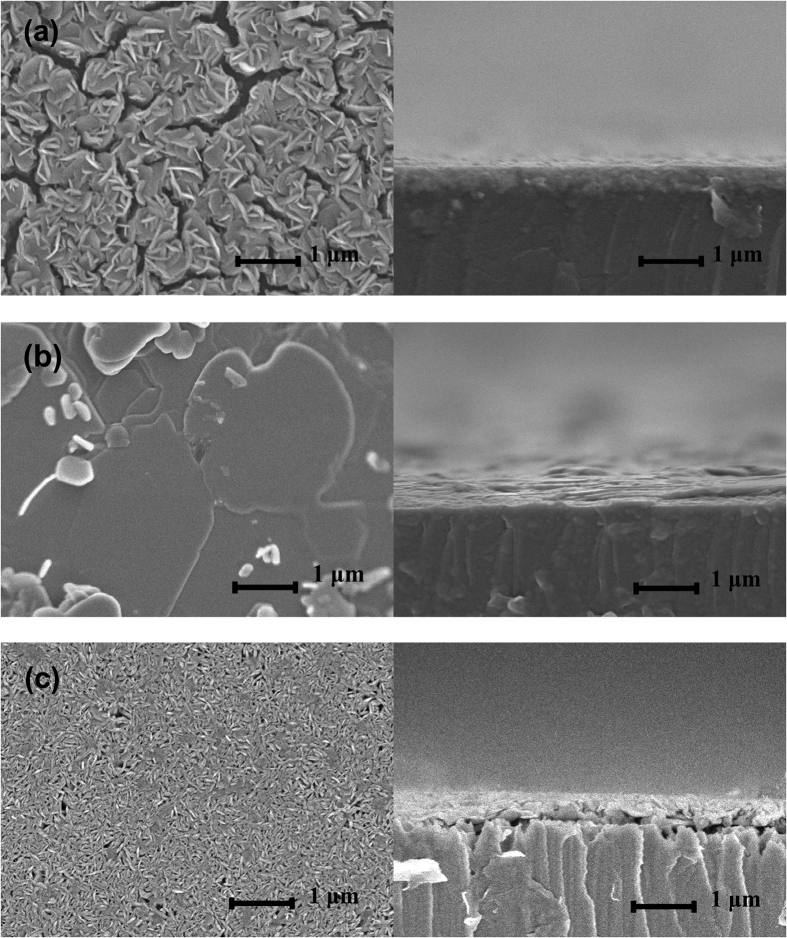
Surface and cross-sectional SEM images of WS_2_ films grown on sapphire substrate (**a**) without Ni promoter (type A film), (**b**) with Ni promoter (type B film) and (**c**) WS_2_ film grown on n-GaN/sapphire substrate with Ni promoter (type C film).

**Figure 3:**
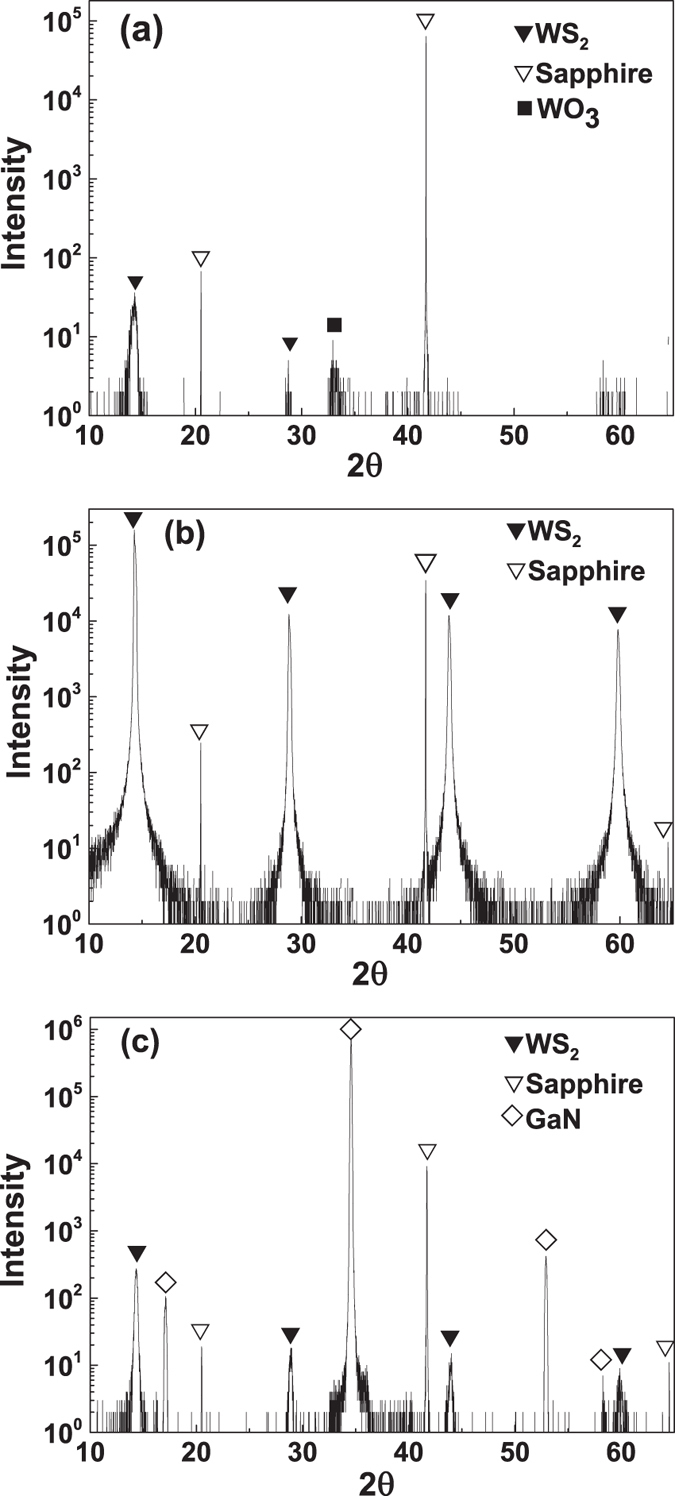
HXRD patterns for WS_2_ grown (**a**) without and (**b**) with Ni promoter on sapphire substrate; (**c**) WS_2_ grown with Ni promoter on n-GaN/sapphire substrate.

**Figure 4:**
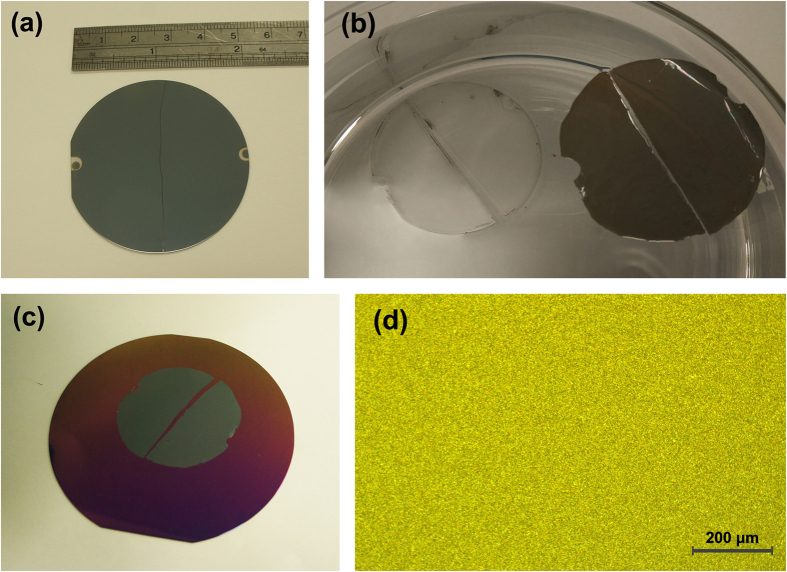
(**a**) The as-grown type B WS_2_ film on 2-inch sapphire wafer; (**b**) separated polystyrene polymer-coated WS_2_ film floated on water; (**c**) 2-inch WS_2_ thin film transferred to a four inch SiO_2_/Si wafer without observable wrinkles, cracks and polymer residues; and (**d**) optical image of WS_2_ after being transferred to the SiO_2_.

In addition to the selection of the proper carrier polymer, it is found that successful wafer-scale transfer is strongly dependent on the film quality and microstructure. As discussed above, type A film exhibited a mixture of type I and type II oriented crystal pieces as shown in Fig. 1a. On the other hand type B film was uniform with large layered crystals with their vdW planes parallel to the substrate in which wafer-scale thin films can be perfectly exfoliated from the growth substrates and transferred to the target substrates. This is because of the vdW planes of the layered crystal structure oriented in parallel to the substrate. This allows the water molecules to uniformly penetrate into the interface between the film and the substrate resulting in the uniform separation of the film. However, it is quite difficult to separate type A films from the growth substrates with acceptable integrity and in most cases the type A films were partially separated from the growth substrates and exhibited substantial concentration of cracks induced during the transfer process. This is attributed to the existence of type I crystallites. Strong interaction between the dangling bonds of type I crystallites and the substrate surface prevents water molecules from penetrating uniformly into the interface between the film and the substrate. Therefore, only type B film can be successfully transferred to n-GaN for the fabrication of p-n junction.

### Fabrication of WS_2_/GaN p-n Junction

**Figure 5:**
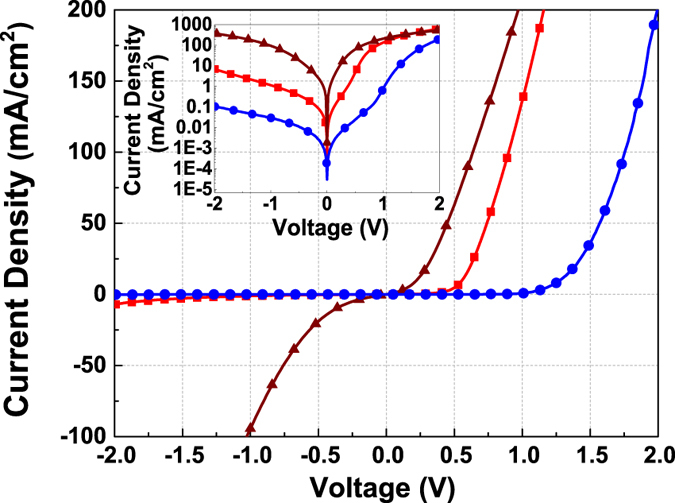
I-V curves of p-type WS_2_ film on n-type GaN p-n junction for: top-transferred device (blue solid circles); back-transferred device (red squares); and direct growth WS_2_/GaN device (brown triangle). The inset shows the I-V plotted in semi-log scale.

To demonstrate the feasibility of the layer transfer process for the fabrication of high quality device, we transferred p-type WS_2_ thin film (type B), both with the back surface (the growth interface) and top surface, onto the n-type GaN substrate to form p-n junctions. The two types of p-n junctions are referred to as back-transferred p-n junction and top-transferred p-n junction respectively. In order to study the impact of back-transferred and top-transferred p-n junction, p-n junction was also fabricated by using type C film, WS_2_ grown directly on n-GaN. As shown in Fig. 5, the *I-V* curves of the transferred p-n junctions demonstrated leakage current densities of 1.15 mA/cm^2^ and 29.6 μA/cm^2^ at −1 V for the back-transferred and top-transferred junction respectively. The respective turn-on voltages for the two devices were 0.5 V and 1.2 V, indicating good interface adhesion of transferred WS_2_ thin film on the GaN substrate. The results compare favorably to the p-n junction formed by direct deposition of WS_2_ on GaN substrate which gives a large leakage current density of 92.4 mA/cm^2^ at −1 V. The experimental results show that the transferred devices exhibit significantly lower leakage current compared to the one fabricated by direct growth of WS_2_ on the n-type GaN layer. This is attributed to the better crystal quality of WS_2_ thin film grown on sapphire substrates compared to that grown directly on GaN substrates as demonstrated by the SEM and XRD results above. The defect density of the WS_2_ film grown directly on GaN is believed to be much higher than the transferred film resulting in significant degradation in the device performance. It is also noted that the leakage current for the top-transferred p-n junction was about two orders of magnitude lower than that of the back-transferred p-n junction due the better quality for the top surface of the WS_2_ film. It is because the initial stage of thin film growth usually generates more step defects by stacking faults of the initial islands and dislocation defects due to the lattice mismatch with the substrate^33^,^34^,^35^. The higher concentration of defects at the growth interface resulted in relatively larger leakage current for the back-transferred p-n junction. The different turn-on voltages for the two transferred p-n junctions is attributed to the different surface properties of the WS_2_ thin films which was characterized by XPS.

**Figure 6:**
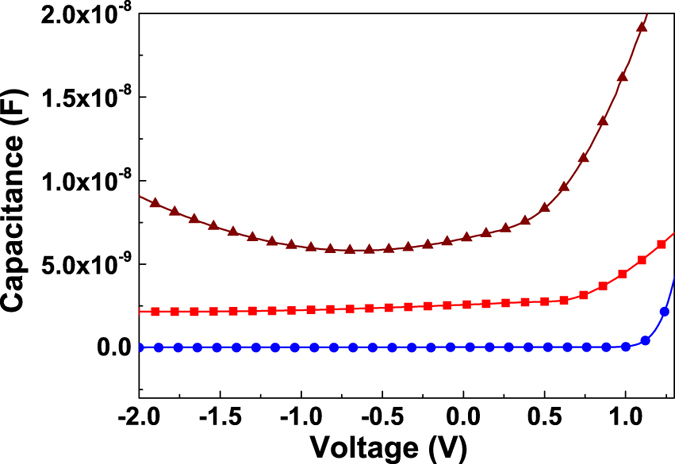
C-V curves of p-type WS_2_ on n-type GaN for: top-transferred device (blue solid circles); back-transferred device (red squares); and direct growth WS_2_/GaN device (brown triangle).

The C-V measurement is consistent with the *I-V* characteristics of the device. Figure 6 shows the C-V curves of the p-n junctions for the three types of devices. The capacitance of p-n junction consists of two components: (i) the space charge capacitance which arises from charge accumulation in the depletion region and is dominant at reverse bias; and (ii) the diffusion capacitance which is associated with the excess carriers and dominates at the forward bias due to the large diffusion current^36^. The capacitance of the transferred p-n junctions decreased with the increase of reverse bias which widens the depletion regions resulting in the decrease of capacitances. The top-transferred p-n junction has a lower capacitance at reverse bias compared to the back-transferred device at the same level consistent with the magnitude of the leakage current. For the as-grown junction, an increase in the capacitance was observed for the biasing voltage below −0.5 V. When the voltage bias was above −0.5 V, the capacitance was dominated by the space charge capacitance and decreased. At voltage below −0.5 V, the capacitance increased with reverse bias indicating the capacitance was dominated by the diffusion capacitance due to the large leakage current induced by the interface and bulk defects. The data show that the high defect concentration for the as-grown device is the main reason for its larger leakage current compared to the transferred device.

**Figure 7:**
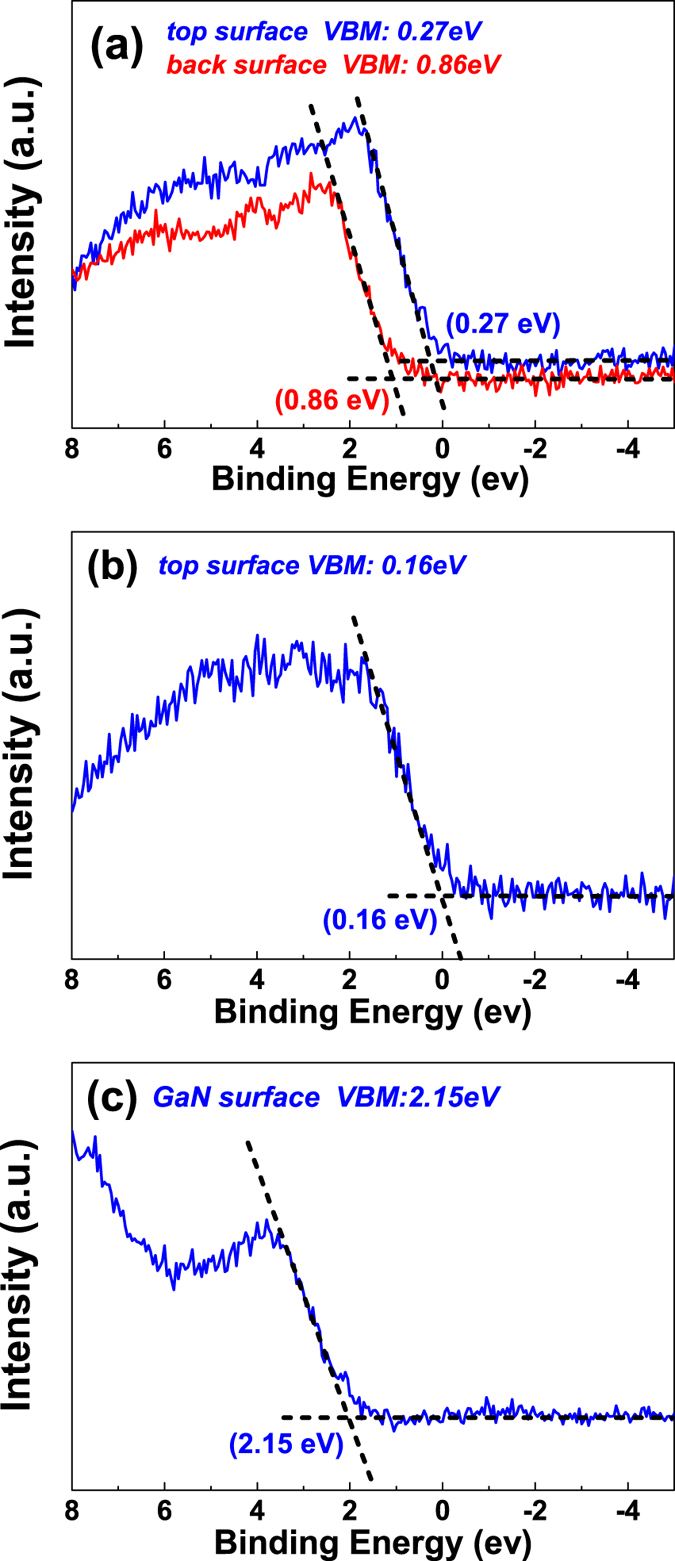
Valence-band XPS spectra of (**a**) the top surface and back surface (growth interface) of WS_2_ grown on sapphire (type B film); (**b**) the top surface of WS_2_ grown on GaN (type C film); and (**c**) GaN film.

**Figure 8:**
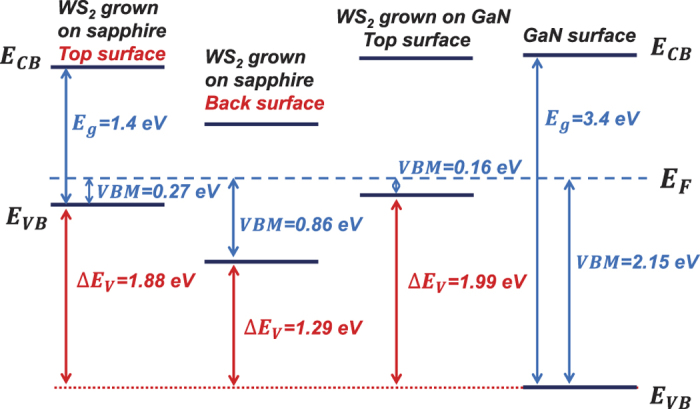
Schematic energy band diagram of the top surface and back surface (growth interface) of WS_2_ thin films grown on sapphire substrate (type B film); the top surface of WS_2_ grown on GaN (type C film); and GaN film.

Figure 7 shows the XPS spectra of the valence band maximum (VBM) for the top surface and back surface (growth interface) of type B film, type C film and GaN film. The C 1 s peak (284.6 eV) was used to calibrate the XPS spectra to compensate the surface charge effect. The position of the VBM with respect to the Fermi level was determined by the intersection between the linear fits to the leading edge of the valence band photoemission and the background. The room temperature band gap of WS_2_ and GaN are found to be 1.4 eV and 3.4 eV respectively^5^,^37^. The band diagrams of these films according to the values obtained from the VBM are shown in Fig. 8. The VBM for the top and back surfaces of type B film are 0.27 eV to 0.86 eV respectively and the film surface changed from p-type to weak n-type. As discussed above, the growth interface of the WS_2_ thin film typically contains more defects compared to the top surface. This explains why monolayer or few layers of WS_2_ ultra-thin film on sapphire are usually n-type due to the existence of S-vacancies which is an n-type defect^34^,^35^,^38^. By the same token, the growth interface of our thick (400 nm) type B film also demonstrated an n-type band diagram. Neglecting the small change in the valence band offset after the transfer of the WS_2_ film onto the GaN substrate, as the interface attraction was dominated by weak vdW force and the surface of WS_2_ and GaN is expected to keep its original electronic property, Δ*E*_*V*_ of the top-transferred p-n junction is 1.88 eV which is about 50% larger than the Δ*E*_*V*_ of the back-transferred p-n junction of 1.29 eV. Larger Δ*E*_*V*_ leads to greater built-in voltage which is consistent with the increase in the turn-on voltage of the top-transferred p-n junctions. The experimental Δ*E*_*V*_ of 1.29 eV and 1.88 eV for the back-transferred and top-transferred devices are well consistent with the change in the turn on voltage from 0.5 V to 1.2 V for the two devices. The larger Δ*E*_*V*_ also contributes to the reduction in the leakage current. The VBM of the top surface of WS_2_ layer deposited on GaN is little higher than WS_2_ grown on sapphire substrate, this shift is attributed to the higher concentration in the bulk defects of poor quality of WS_2_ grown on GaN substrates.

## Conclusions

We have reported on the growth of high quality p-type WS_2_ thin film with the carrier mobility as high as 63.3 cm^2^/Vs by CVD method using Ni as the texture promoter. Our results demonstrate that using the Ni texture promoter, the microstructure of the WS_2_ film changed from randomly orientated crystallites to large layered crystals parallel to the substrate with strong preferential growth along the [001] direction. Wafer-scale WS_2_ thin films grown with Ni texture promoter were exfoliated and transferred onto arbitrary flat substrates by the etching-free transfer technique without any observable wrinkles, cracks, or polymer residues. The etching-free transfer method utilizing different surface properties for the WS_2_ film and the sapphire substrate and does not involve the use of any corrosive chemical etchants. As a result the transferred film demonstrated high level of film integrity that facilitates the fabrication of good quality p-n junctions and the recycling of the substrates. The selection of the carrier polymer and the film’s quality and microstructure are two key points for the success of the wafer-scale transfer of the WS_2_ thin film. The p-n junction fabricated by transferring the p-type WS_2_ onto n-type GaN had a small leakage current density of 29.6 μA/cm^2^ at −1 V, while the direct growth WS_2_/GaN p-n junction exhibits large leakage current density of 92.4 mA/cm^2^ at −1 V. This is attributed to the degradation of the GaN layer at the high growth temperature of 1000 °C resulting in poor crystal quality of the WS_2_ film grown directly on the GaN substrates. The leakage current of top-transferred p-n junction was much lower than that of the back-transferred p-n junction due the better quality of top surface of the WS_2_ film. The VBM of top surface and back surface (growth interface) of WS_2_ film grown on sapphire with Ni promoter changed from 0.27 eV to 0.86 eV and the film surface changed from p-type to weak n-type. It is believed that this etching-free exfoliation method will greatly expand the applications of WS_2_ thin films in electronic and optoelectronic devices. Furthermore, the technique should also be applicable to other material systems such as MoS_2_, WSe_2_, SnS_2_, etc.

## Methods

### Preparation of WS_2_ Thin Film

Three types of WS_2_ films were grown at the high temperature of 1000 °C by CVD technique using identical conditions except: i.) WS_2_ film grown on sapphire substrate without using the Ni texture promoter (type A); ii.) WS_2_ film grown on sapphire substrate with the use of a 5 nm thick Ni texture promoter (type B); and iii.) WS_2_ film grown on n-type GaN thin films with the use of a 5 nm thick Ni texture promoter (type C). For the growth of type A samples, the sapphire substrates were first cleaned using standard cleaning procedure prior to the deposition of a WO_3_ layer by e-beam evaporation. For type B samples, a 5 nm thick Ni texture promoter was deposited by e-beam technique prior to the deposition of the WO_3_ layer which is followed by the growth of the WS_2_ layer inside the quartz tube. For type C samples, we first deposited n-type GaN thin films on (0001) sapphire substrates by metalorganic chemical vapor deposition technique^39^ followed by the e-beam evaporation of 5 nm thick Ni layer and the WO_3_ layer. The thickness of the WO_3_ precursor layer was 400 nm and was monitored by an INFICON *in-situ* quartz crystal thickness monitor. The actual film thickness was also measured *ex-situ* by a surface profiler. The samples were then inserted into the quartz tube for the growth of WS_2_ layers. The substrates and the sulfur source were located at different positions within the quartz tube (Supplementary Fig. S3). A quartz boat with sulfur pieces (5 g, 99.998%, Sigma-Aldrich) was located on the upstream to the substrates. A heating belt, wrapping around the quartz tube, was used to heat the sulfur sources. The WO_3_ film was placed in a downstream location. Ultra-high purity Ar gas with a flow rate of 100 sccm was used as the carrier gas during the growth process. The pressure in the quartz tube was reduced to 5 × 10^−2^ torr and the temperature of the substrate was raised to 1000 °C while the sulfur source was kept at 130 °C using the heating belt for 6 hours to ensure the complete reaction of WO_3_ with the S vapor. The temperature profile of the CVD process can be found in the Supplementary Fig. S4. The sample was then allowed to cool to room temperature within the furnace at a rate of 3 °C/min. The thickness of the WS_2_ thin film was about 400 nm, determined by a surface profiler.

### Transfer of WS_2_ Thin Film

We have adopted an etching-free transfer approach for wafer-scale exfoliation and transfer of WS_2_ thin films onto arbitrary flat substrates without observable wrinkles, cracks, or polymer residues. The etching-free transfer method takes advantage of the different surface properties of the WS_2_ thin film and the sapphire substrate. The hydrophobic property of WS_2_ thin film and hydrophilic property of sapphire substrate induced water molecules to penetrate into the WS_2_-sapphire interface resulting in the separation of the WS_2_ layer from the sapphire substrate. The selection of the carrier polymer and the film quality and microstructure (type-I or type-II textured) are the two most important factors in the success of wafer-scale transfer of the WS_2_ thin film. Our findings showed that type II textured WS_2_ is essential for wafer-scale etching-free exfoliation process. WS_2_ consisted of type I textured crystallites cannot be exfoliated because the strong interaction between the type I textured crystallites and the substrate prevented the penetration of water molecules at the interface. This approach, without using any corrosive etchants, enables to reuse of the sapphire substrates. For a normal transfer process, polystyrene polymer solution was spin-coated, at a speed of 4000 rpm for 30 seconds, on the WS_2_ thin film. The sample was then baked at 80 °C for 10 min to dry the polymer. A cut was applied to the polymer surface using a sharp blade along the edge to allow water to penetrate between the polystyrene-coated WS_2_ film and the substrate. The sample was dipped into a deionized water bath for several minutes resulting in the complete separation of the polymer-coated WS_2_ layer from the sapphire substrate. Using this technique, the assembled thin film of polymer coated type II textured WS_2_ was exfoliated from the sapphire substrate and subsequently transferred to a target substrate. Compared to the ordinary transfer of graphene or monolayer TMDCs which only allow the back surface (the growth interface) transfer onto the target substrate, our technique allows transfer of the WS_2_ thin film with both the back surface and the top surface onto the target substrate. For the back surface transfer, the polymer-coated WS_2_ thin film was picked up by the target substrate followed by a gentle baking at 70 °C for 30 minutes to remove the water residue. Then a hard baking at 130 °C for 30 minutes was applied to slightly melt the polymer and spread the film. Finally, the polystyrene polymer was removed from the WS_2_ layer by dipping the sample in toluene for several minutes. For the top surface transfer, the polymer-coated WS_2_ thin film was dipped into toluene for several minutes to remove the polystyrene polymer. The free-standing WS_2_ film was tough enough to keep the film integrity for rollover in the toluene. Then the overturned WS_2_ film was transferred with a Teflon mesh plate into acetone, IPA and DI water in sequence to wash away the solvent. Finally, the free standing WS_2_ thin film was picked up using the target substrate followed by baking at 70 °C for 30 minutes and 130 °C for 30 minutes to secure good adhesion.

### Characterization

The WS_2_ thin film surface morphology was characterized by scanning electron microscopy. A JEOL 6490 microscope was used to take SEM image for the investigation of the film microstructure using an accelerating voltage of 30 kV. The thin film crystal structure was analyzed by high resolution X-ray diffraction using a Rigaku Smartlab 9 kW X-ray diffractometer, employing Cu-K_α1_ radiation source (λ = 1.5406 Å) accompanied by a two-crystal Ge (220) two-bounce hybrid monochromator. Phonon behaviors were investigated by Raman microscope of backscattering configuration with excitation wavelengths of 488 nm. The XPS measurements were carried out on a SKL-12 spectrometer equipped with Al K_α_ X-ray radiation source. C 1 s peak (284.6 eV) was used for calibrating the XPS spectra to compensate the surface charge effect.

### Fabrication of WS_2_/GaN p-n Junction

To examine the feasibility of the etching-free transfer method for the fabrication of heterojunctions we have transferred type B WS_2_ films, grown by vdWR technique, onto Si-doped n-type GaN substrates. The doping concentration of the GaN is about 3 × 10^18^ cm^−3^. This is compared to type C devices in which the p-type WS_2_ layer was grown directly onto the n-GaN layer by vdWR utilizing Ni as the texture promoter. An e-beam evaporated Ni/Au (5 nm/300 nm) bi-layer was used for the fabrication of ohmic contact on the top surface of the WS_2_ layer, and an Al layer (300 nm) was deposited on the back surface of the WS_2_ layer to form ohmic contact.

## Additional Information

**How to cite this article**: Yu, Y. *et al.* Fabrication of WS_2_/GaN p-n Junction by Wafer-Scale WS_2_ Thin Film Transfer. *Sci. Rep.* **6**, 37833; doi: 10.1038/srep37833 (2016).

**Publisher's note:** Springer Nature remains neutral with regard to jurisdictional claims in published maps and institutional affiliations.

## Supplementary Material

Supplementary Information
