# Supplementary material for: Fabrication of WS2/GaN p-n Junction by Wafer-Scale WS2 Thin Film Transfer
Source: Sci Rep. 2016 Nov 29;6:37833. doi: 10.1038/srep37833 (PMC5126671; doi:10.1038/srep37833)
Supplement: Supplementary Information [file srep37833-s1.pdf]

Supplementary Information

Fabrication of WS_2/GaN p-n Junction by Wafer-Scale WS_2 Thin Film Transfer

Yang Yu[1], Patrick W. K. Fong[1], Shifeng Wang[1], and Charles Surya*[1]
[1]Department of Electronic and Information Engineering
The Hong Kong Polytechnic University
Hong Kong, China
E-mail: charles.surya@polyu.edu.hk

Figure S1 shows the Raman spectrum of that the A_{1g} mode located at 421.8 cm^{-1} represents an out-of-plane vibration for the S atoms whereas the E_{2g}^{1} mode located at 356.9 cm^{-1} comprises the in-plane displacement of W and S atoms, and the second-order phonon mode is labeled as the 2LA.[1-2] The FWHM of A_{1g} mode for type A film is 4.03 cm^{-1}, which is 52% wider than the FWHM of A_{1g} mode for type B film which is 2.65 cm^{-1}. The Raman intensity ratio of $I(E_{2g}^{1})/I(A_{1g})$ for type B film is 0.617, which is reduced to 0.454 for type C film, as shown in Figure S2, implying the in-plane vibration is decreased compared to the out-of-plane vibration, which should be due to the sub-grain disorientation or smaller-sized crystallites of the WS_2 film grown on GaN substrate.

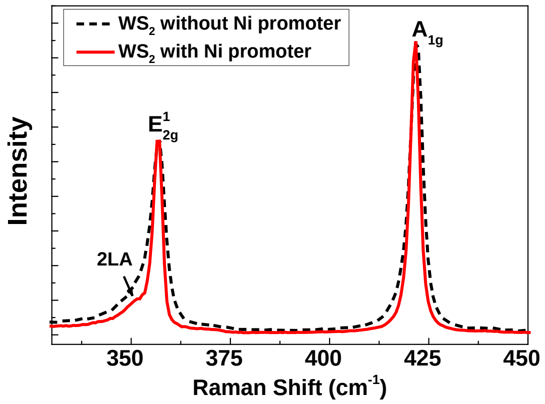

Figure S1. Raman spectrum of WS_2 grown without (black dash line) and with (red solid line) Ni promoter on sapphire substrate

.

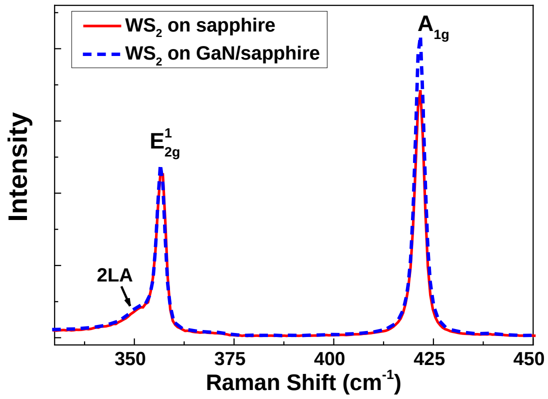

Figure S2. Raman spectrum of WS_2 grown on sapphire substrate (red solid line) and on GaN/sapphire substrate (blue dash line) with Ni promoter.

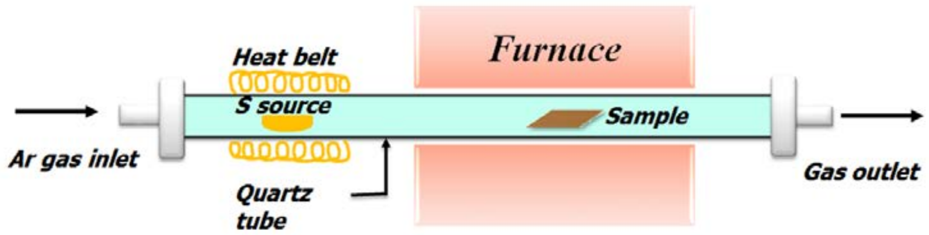

Figure S3. Schematic diagram of the experimental setup used for the growth of WS_2 thin films.

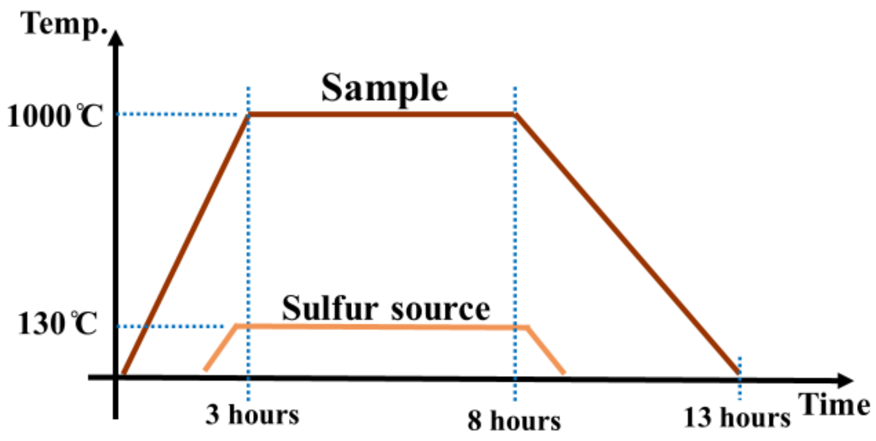

Figure S4. Temperature profile of the CVD process.

Figure S5 is the schematic diagram showing the etching-free wafer-scale transfer process for type II WS_2. It is noted that type II WS_2, with the vdW planes parallel to the substrate, is a key factor in the success of wafer-scale transfer. Type I WS_2, with its vdW planes perpendicular to substrate, prevents the water molecules to penetrate evenly into the film-substrate interface.

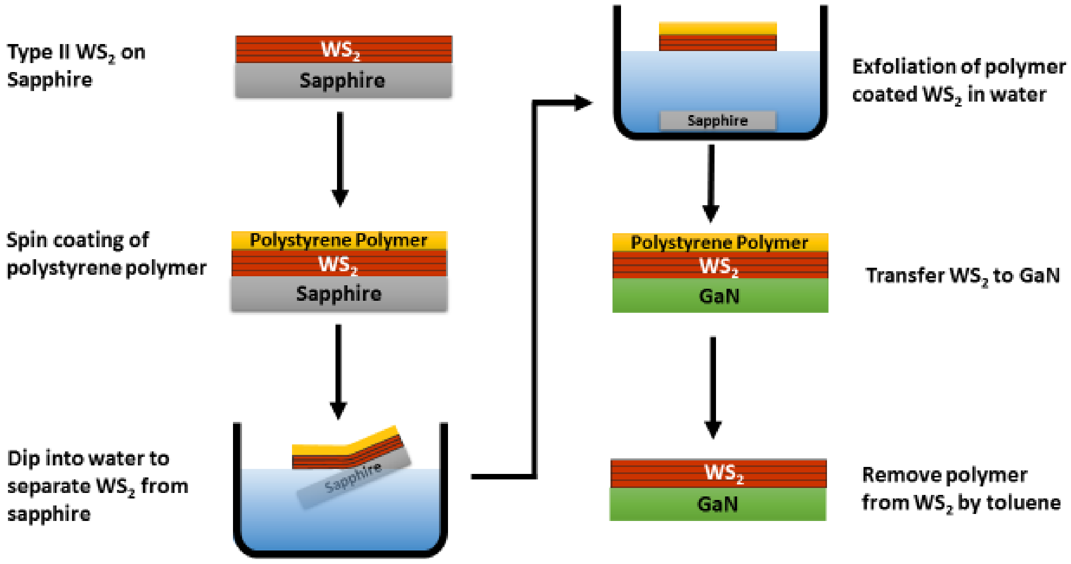

Figure S5. Schematic diagram of the etching-free transfer of type II WS_2.

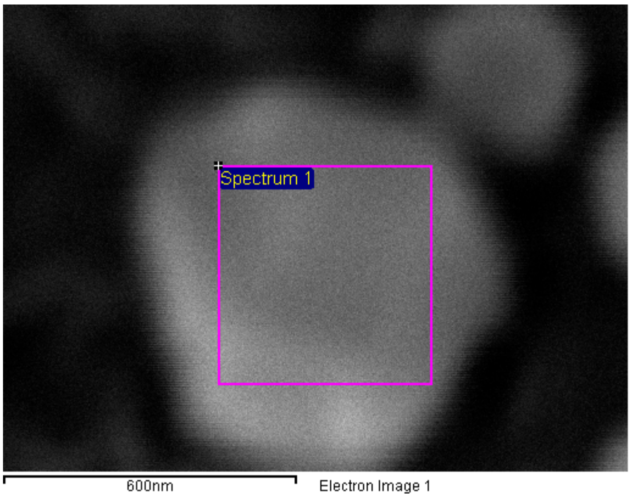

Figure S6. EDX analysis on a 500nm NiS_x residue.

Table S1. Summary of the EDX analysis on a 500nm NiS_x residue.

Element	Weight%	Atomic%
C K	11.79	38.69
Al K	12.25	17.90
S K	19.97	24.55
Ni K	15.85	10.64
W M	13.32	2.86
Au M	26.82	5.37
Totals	100.00	

Reproducibility of turn-on voltage of the p-n junctions can be well controlled by our transfer fabrication method, and the oscillations the carrier mobility of WS_2 thin film were at an acceptable level. 3 group data of carrier mobility of WS_2 thin film at the same growth condition and turn-on voltage of 3 different kind of p-n junctions are listed in the Table S2 and Table S3.

Table S2. The carrier mobility of three groups of WS_2 thin films at the same growth condition.

WS_2 thin film with Ni promoter	Carrier mobility [cm^2/Vs]
1	*63.3*
2	*58.7*
3	*60.1*

Table S3. The leakage current and turn-on voltage of three groups of back-transferred, top-transferred and as-grown p-n junctions.

Back-transferred p-n junction	Leakage current [mA/cm^2]	Turn-on voltage [V]
1	*1.15*	*0.50V*
2	*2.88*	*0.45V*
3	*3.75*	*0.51V*
Top-transferred p-n junction	Leakage current [mA/cm^2]	Turn-on voltage [V]
1	*0.0296*	*1.20V*
2	*0.0971*	*1.07V*
3	*0.0187*	*1.22V*
As-grown p-n junction	Leakage current [mA/cm^2]	Turn-on voltage [V]
1	*92.4*	*0.17V*
2	*80.6*	*0.16V*
3	*73.8*	*0.17V*

References

1. Zhao, W. J. *et al.* Lattice dynamics in mono- and few-layer sheets of WS_2 and WSe_2. *Nanoscale* **5,** 9677-9683 (2013).
2. Sourisseau, C., Cruege, F., Fouassier, M. & Alba, M. Second-order Raman effects, inelastic neutron scattering and lattice dynamics in 2H-WS_2. *Chem. Phys.* **150,** 281-293 (1991).
